# Deep Tissue Injury in Development of Pressure Ulcers: A Decrease of Inflammasome Activation and Changes in Human Skin Morphology in Response to Aging and Mechanical Load

**DOI:** 10.1371/journal.pone.0069223

**Published:** 2013-08-14

**Authors:** Olivera Stojadinovic, Julia Minkiewicz, Andrew Sawaya, Jonathan W. Bourne, Peter Torzilli, Juan Pablo de Rivero Vaccari, W. Dalton Dietrich, Robert W. Keane, Marjana Tomic-Canic

**Affiliations:** 1 Department of Dermatology & Cutaneous Surgery, Wound Healing and Regenerative Medicine Research Program, University of Miami Miller Medical School, Miami, Florida, United States of America; 2 Department of Physiology & Biophysics, University of Miami Miller Medical School, Miami, Florida, United States of America; 3 Tissue Engineering, Regeneration and Repair Program, Hospital for Special Surgery, New York, New York, United States of America; 4 Departments of Neurological Surgery, The Miami Project to Cure Paralysis, University of Miami Miller School of Medicine, Miami, Florida, United States of America; Université de Technologie de Compiègne, France

## Abstract

Molecular mechanisms leading to pressure ulcer development are scarce in spite of high mortality of patients. Development of pressure ulcers that is initially observed as deep tissue injury is multifactorial. We postulate that biomechanical forces and inflammasome activation, together with ischemia and aging, may play a role in pressure ulcer development. To test this we used a newly-developed bio-mechanical model in which ischemic young and aged human skin was subjected to a constant physiological compressive stress (load) of 300 kPa (determined by pressure plate analyses of a person in a reclining position) for 0.5–4 hours. Collagen orientation was assessed using polarized light, whereas inflammasome proteins were quantified by immunoblotting. Loaded skin showed marked changes in morphology and NLRP3 inflammasome protein expression. Sub-epidermal separations and altered orientation of collagen fibers were observed in aged skin at earlier time points. Aged skin showed significant decreases in the levels of NLRP3 inflammasome proteins. Loading did not alter NLRP3 inflammasome proteins expression in aged skin, whereas it significantly increased their levels in young skin. We conclude that aging contributes to rapid morphological changes and decrease in inflammasome proteins in response to tissue damage, suggesting that a decline in the innate inflammatory response in elderly skin could contribute to pressure ulcer pathogenesis. Observed morphological changes suggest that tissue damage upon loading may not be entirely preventable. Furthermore, newly developed model described here may be very useful in understanding the mechanisms of deep tissue injury that may lead towards development of pressure ulcers.

## Introduction

Pressure ulcers (PU), defined as breaks in the integument caused by continuous pressure of the body weight to skin have been implicated as one of the most frequent causes of death in elderly, wheelchair and bed–bound individuals. As many chronic wound types, PU is multifactorial disease. Multiple physical factors lead to the development of PU including: static pressure or stress, shearing forces, friction, and moisture, but these alone are insufficient to produce tissue damage resulting in a PU. However, if these factors are combined with host-specific factors such as immobility, aging, neurologic disease, incontinence and malnutrition, a PU may form.

Prolonged load/pressure in conjunction with tissue ischemia is believed to play a major role in PU development. A PU can develop in as little as 2 hours of immobility [1], [2]. Initial changes, observed as deep tissue injury (DTI), can develop in patients even during prolonged surgeries [1], [3]. The National Pressure Ulcer Advisory Panel reports wide ranges of prevalence among patients in the United States estimated to be 1.3 to 3 million [4]. Approximately 2.5 million PU require treatment annually, representing the second most frequent cause for hospital readmissions [5], with estimated costs of hospital-acquired PU at $2.2 – $3.6 billion [6]. Federal Government issued guidelines for hospitals and care-takers for risk assessment, documentation and prevention because PUs are the source of numerous complications and often result in multiple hospitalizations [7]. Due to limited knowledge regarding the molecular pathogenesis of these ulcers there is no treatment currently approved by FDA as efficacious.

The challenge in studying the development of PU results from lack of adequate experimental models that accurately resemble human disease. Current knowledge originates mostly from analyses of human tissue, wound fluid and a few animal models. Reports from studies showed elevated levels of IL1, TNF, MMP's and defensins in PU [8]–[15]. Animal studies suggest synergistic effects of age and ischemia as contributing factors [16]. Study conducted to assess the effect of prolong mechanical load using bioengineered skin showed involvement of pro-inflammatory mediators [17], [18].

Keratinocytes participate in innate immune signaling and are the first responders to the danger signals by secreting pro-inflammatory cytokines, namely interleukin-1β (IL1-β) [19], [20]. The inflammasome, a cytosolic, multiprotein platform that activates pro-inflammatory caspases and IL-1β is expressed in human keratinocytes both *in vitro* [21] and *in vivo* [22], [23]. It is activated by diverse molecular patterns released from stressed and damaged cells [24]–[26].

Here, we report development of a novel experimental pressure model utilizing a Mechanical Explant Test System (METS) [27], [28] to assess the effect of load and ischemia on human skin explants of different age. To the best of our knowledge, this report is the first experimental approach aiming to determine specific changes that occur in human skin due to static pressure in conjunction with ischemia. We found that the morphology of skin differed among young and aged individuals in response to load. We observed subepidermal separation and a change in collagen alignment in aged skin in response to load. We also report that young skin has significantly higher levels of inflammasome proteins than aged skin. Loading young skin resulted in a rapid increase in inflammasome proteins levels that was maintained for 4 hours. In contrast, no significant alteration in inflammasome protein expression was found in loaded aged skin. Taken together, our findings support the idea that the effects of aging and load synergize to contribute to morphological changes that lead to development of DTI and further to PU. Not only morphological changes occurred more rapidly in aged skin but no change in inflammasome protein expression was found in aged skin, underscoring the role of aging as an important factor that influences the innate inflammatory response and may contribute to the pathogenesis of PU.

## Materials and Methods

### Pressure Plate Measurements

In vivo skin contact stress measurements were made on a male subject weighing 183 pounds/83 mg (813.4 Newtons). This portion of the study was found to be exempt under 45 CFR46.101.2 by the IRB at the Hospital for Special Surgery. The written consent was obtained during original data collection during a routine educational demonstration and data in our present study did not contain any of the 18 identifiers noted in the privacy rule and as such no further consent was needed. The subject was seated on a flat surface and positioned on the approximate center of an EMED-X pressure sensor array platform (Novel, Munich, Germany). The sensor array was embedded flush with the surrounding surface and consisted of 6,080 force transducers at a resolution of four sensors per cm^2^, a total sensor area of 1,520 cm^2^ and measuring 47.5 cm by 32.0 cm [29]. Pressure measurements were recorded for 30 second intervals and the recorded pressure measurements were analyzed using standard EMED software (model EMED X/R®, version 13.3.50, Novel, Munich, Germany and St. Paul, MN) [29].

### Specimen Loading and Tissue Processing

After IRB approval, skin specimens were obtained from 6 Caucasian female patients undergoing abdominoplasty within 15 minutes post-surgery were grouped based on age into: young (29–35 years old) and aged (54–60 years old) groups. The skin was cleaned of blood and fat and ten full thicknesses, 7 mm biopsies were obtained per skin specimen.

We have used previously described Mechanical Explant Test System (METS) [27] to apply load to 7 mm full thickness skin specimens. A flat porous platen was used to equally distribute the load over the entire specimen, producing a uniaxial static compressive stress (hydrostatic pressure) throughout the specimen. The METS applied a static stress of 300 kPa for 0.5, 1, 2 and 4 hrs. Unloaded skin maintained at the air-liquid interface served as control for each corresponding time point. Samples were either snap frozen or 10% formalin fixed, processed and paraffin embedded. 8 µm sections were subjected to hematoxylin and eosin staining (H&E) and picrosirius red staining using previously described protocol [20], [30], [31].

### TUNEL and Collagen Alignment Assays

De-paraffinisation and rehydration were carried out as previously described [32]. TUNEL assay was performed using kit (GenScript USA Inc), following commercial protocol. The slides were mounted using Fluorescein-FragEL™ Mounting Media. Digitally captured picrosirius red stained images of sections were analyzed with ImageJ (version 1.47; http://rsbweb.nih.gov/ij/) using a protocol adapted from Noorlander et al. [33]. Briefly, images were processed using a binary filter, resulting fiber outlines were then fitted with ellipses, then mean length of the major axis was then determined as a measurement for orientation of the bundles of aligned collagen fibers in the plane of the section [33].The mean value of this length parameter was based on three images per section, with a total of 15 specimens per age group, in three serial sections of a specimen was used as a collagen alignment index; the collagen alignment indices of control and loaded skin were compared and differences were analyzed.

### Immunobloting

Proteins were extracted using Tissue-PE LB Kit (Geno technology, inc., MO) with addition of Protease (Sigma-Aldrich, MO) and Phosphatase inhibitor Cocktail Set III (Calbiochem, Ca) according to the manufacturer protocols. Protein lysates were mixed with 5× SDS Laemmli Sample Buffer (Sigma Aldrich, St. Louis, MO) and then resolved in 10–20% Tris-HCl Criterion precasted Gels (Bio-Rad, Hercules, CA), transferred to polyvinylidene difluoride membranes (Applied Biosystems, Foster City, CA) and placed in blocking buffer for 1 hour (Applied Biosystems) Membranes were incubated with the primary antibodies (1∶500) against NLRP3 (Enzo Life Sciences, Farmingdale, NY), ASC (kindly provided by Dr. Robert Keane), caspase-1 (Imgenex, San Diego, CA) and IL-1β (Cell Signaling, Danvers, MA), washed in blocking buffer and incubated with either rat or mouse secondary horseradish peroxidase (HRP)-linked antibodies (Cell Signaling, Danvers, MA). Proteins were visualized by chemiluminescence with a phototope-HRP detection kit (Cell Signaling Danvers, MA). Immunoblots were stripped with Restore, Western Blot Stripping Buffer (Pierce, Rockford, IL), and blotted using mouse monoclonal anti-β-actin antibody (1∶5000; Sigma, St. Louis, MO) to test protein loading. The band densities were quantified with UN-SCAN-IT software, and all data were normalized to β-actin.

## Results

### Development of *ex vivo* human model to study deep tissue injury in response to static pressure

**Figure 1:**
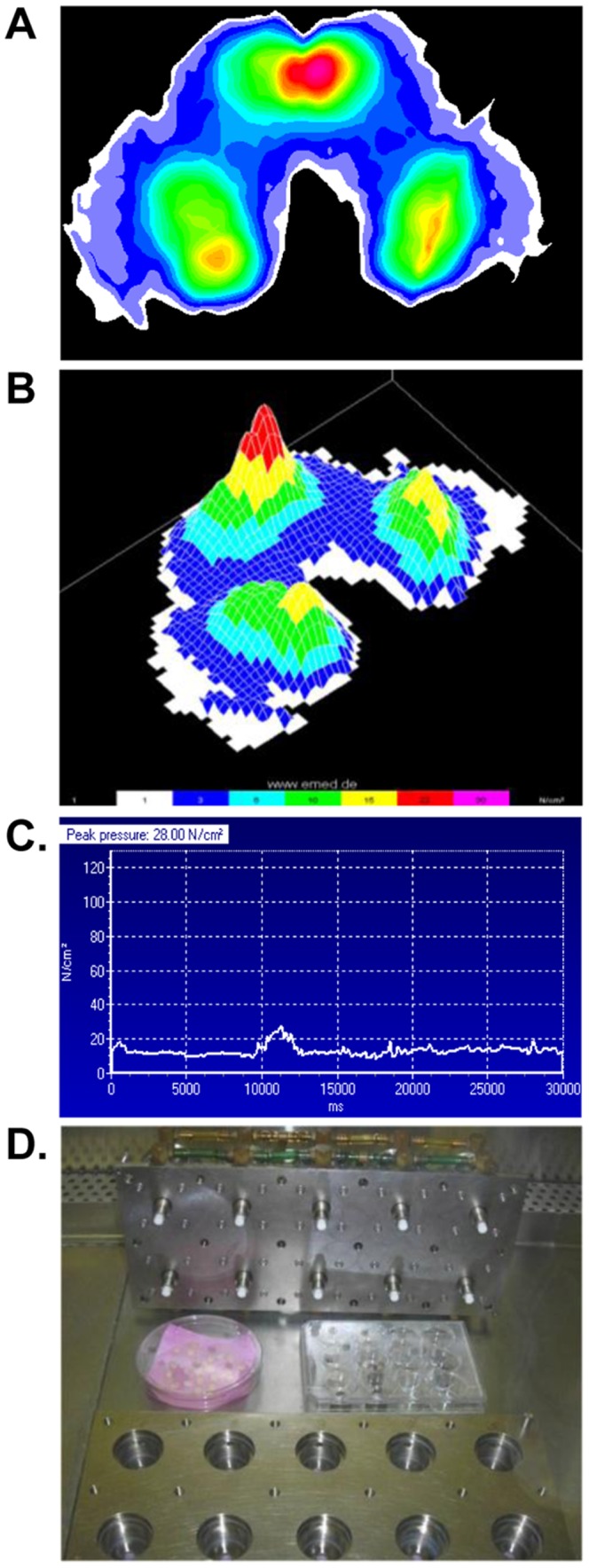
Maximum contact pressure measurements. An EMED-X sensor array was used to measure contact pressure with sub-cm^2^ resolution on a seated individual. A maximum pressure map is shown in the horizontal view (**A**) and rotated to illustrate the spatial pressure distribution in 3-D (**B**). The graph shows the pressure in N/cm^2^ (1 N/cm^2^ = 10 kPa) as a function of time for the region of peak pressure(**C**). METS system used for skin loading (**D**).

We used a pressure sensor array to directly measure skin contact pressure. By taking direct measurements on a subject placed in a reclined position we determined *in vivo physiological* contact surface area and, applied force, and these were used to calculate the contact stress/pressure at sub-cm^2^ spatial resolution. This allowed us to quantify and visualize the peak pressure and pressure variations across the subject's contact with the flat plate using the EMED-X pressure sensor system (**Figure 1**). The maximum peak pressure measured was 280 kPa as illustrated by the contact pressure heat maps (**Figure 1A, B, C**). The point of maximum pressure occurred below the sacrum with additional regions of elevated pressure below the ischial tuberosities. Based on *in vivo* animal data [11], [34] and our own measurements with the pressure sensor arrays, we decided to apply a static 300 kPa stress for the purpose of the present study.

To assess changes in tissue morphology in response to load we utilized previously developed METS system [27], [28] to load a skin in confined, uniaxial static compression of 300 kPa. Skin derived from young and aged individuals was loaded for 0.5, 1, 2 and 4 hrs (**Figure 1D**). We chose these particular time points due to evidence indicating that the frequency and intervals between turning a patient may be more critical than pressure magnitude in the development of PU. In fact, it has been suggested to turn patients every 2 hrs, and this practice remains the basis of prevention strategies since 1959 [35], [36]. Therefore we aimed to study changes in shorter time-span (0.5 and 1 hr), suggested time (2 hrs) and prolonged load (4 hrs). Furthermore, skin explants utilized in this model do not have a vascular supply, and thus mimicked ischemic conditions.

### Tissue morphology reveals differences between young and aged skin 2 and 4 hours after exposure to uniaxial static compressive stress (load/pressure)

To characterize skin changes we employed routine H&E staining and analyzed skin of young (avg.32.5 y.o) and aged (avg. 57.5 y.o) individuals in response to load. Loaded samples were compared to unloaded, control skin kept under the same conditions for an equal duration. Thus, each sample pair, loaded and unloaded, originated from the same individual. In addition, to minimize diversity we analyzed only Caucasian female patients and specific skin location. We did not observe morphological differences in the epidermis of both age groups in response to load. Keratinocytes in control and loaded skin did not show necrotic keratinocyte cell death preceding or following skin loading at any time point.

**Figure 2:**
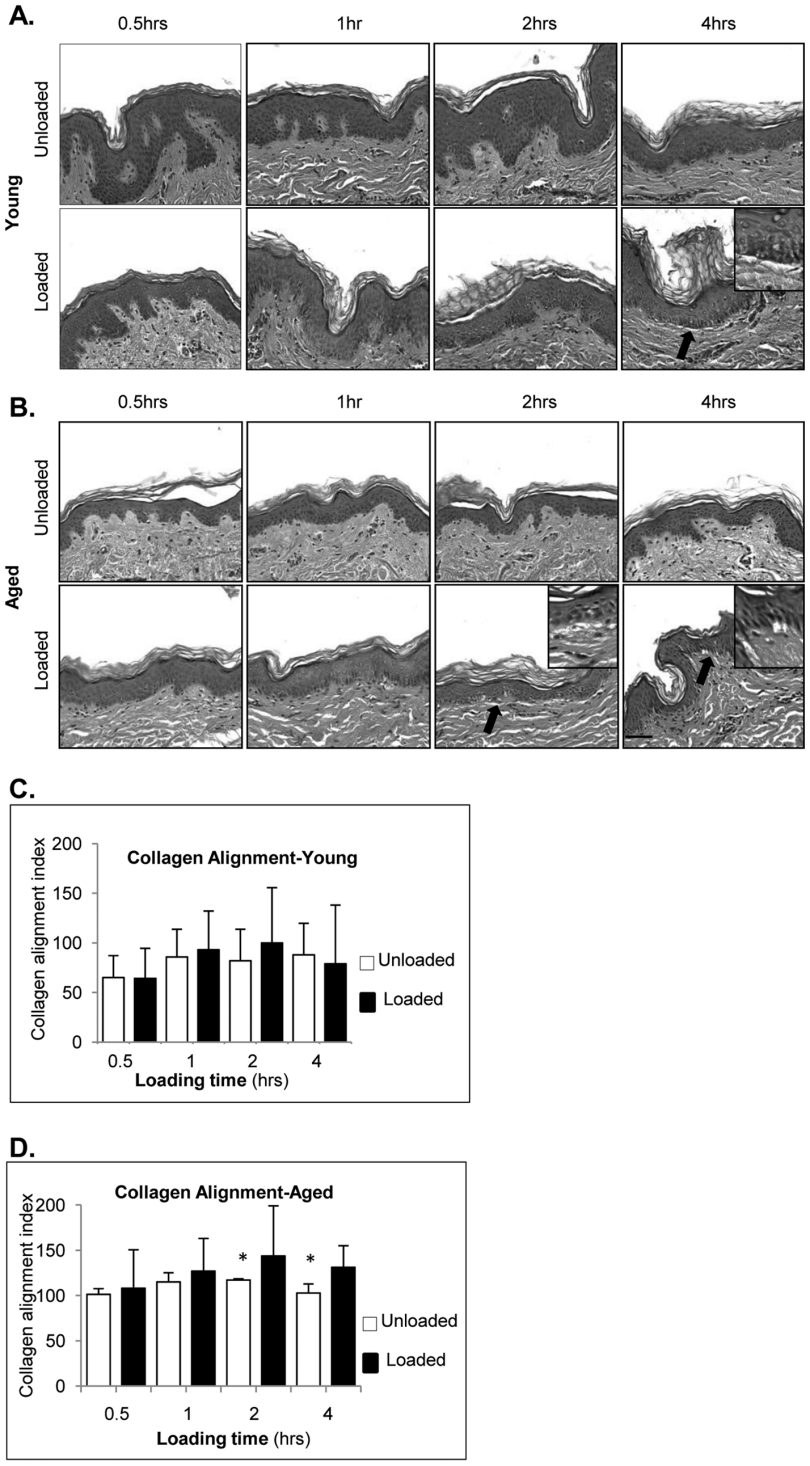
Loading induces sub-epidermal separation and alters orientation of collagen fibers in aged individuals. H&E staining of unloaded and loaded young (**A**) and aged (**B**) skin for 0.5, 1, 2 and 4 hrs (n = 15 specimens per n = 3 experimental replicates). Breaks in a near proximity to basement membrane, as indicated by black arrows, are observed 4 hrs after loading in young and 2 and 4 hrs after loading in aged skin. Inserts represent enlarged images of the areas indicated by black arrows. Magnification 20×. Scale bar 100 µm. Orientation of collagen fibers in a dermis of unloaded and loaded young (**C**) and aged (**D**) skin. Quantification of collagen orientation in a plane of section indicates significantly higher mean value in loaded aged skin after 2 and 4 hours of loading. Values are expressed as mean ± SD (n = 15 specimens per n = 3 experimental replicates). * Indicates p<0.05 by paired Student t-test.

To determine if loading induced apoptosis in epidermal keratinocytes we utilized TUNEL assay. We did not observe double-stranded DNA fragmentation, TUNEL positive cells, in any tested specimens or time points (data not shown). However, we observed subepidermal separation in both young and aged skin but at the different time points (**Figure 2 A, B**). These subepidermal separations were seen consistently in skin from all tree donors. The extent of sub-epidermal separation showed variability among loaded biopsies. Morphological changes were found in dermis of young skin upon 4 hrs of loading (**Figure 2A**). Interestingly, similar changes occurred in aged skin at earlier time point, 2 hrs, and were maintained at 4 hrs (**Figure 2B**), suggesting that aged skin is more susceptible to morphological changes due to applied load and present ischemia than the skin derived from young individuals.

### Two hours of load alters orientation of collagen fibers in dermis of aged skin

Collagen fiber orientation was evaluated using picosirius red staining viewed with polarized light microscopy. To further analyze changes occurring in dermis of young and aged individuals in response to loading we utilized a quantitative microscopic method to assess changes in the orientation of collagen fibers using a protocol adapted from Noorlander et al. [33]. To quantitatively determine the orientation of collagen fibers in the dermis we converted digital images of sections into binary images and analyzed them on the basis of the measured length of the collagen fibers in the plane of the section as a measure for the fibers orientation [33]. The orientation of collagen fibers in sections of aged skin loaded for 2 and 4 hrs differed significantly when their length was measured compared to control, unloaded, aged skin (**Figure 2, C, D**). The earliest detectable changes observed in aged skin were found at 2 hrs of loading (**Figure 2D**). In contrast, no significant changes were found in young skin (**Figure 2C**). These results indicate that aged skin might be more susceptible to changes in collagen alignment in response to prolonged load and ischemia.

### Aged skin shows no detectable levels of active IL-1β, which is not regulated by load

**Figure 3:**
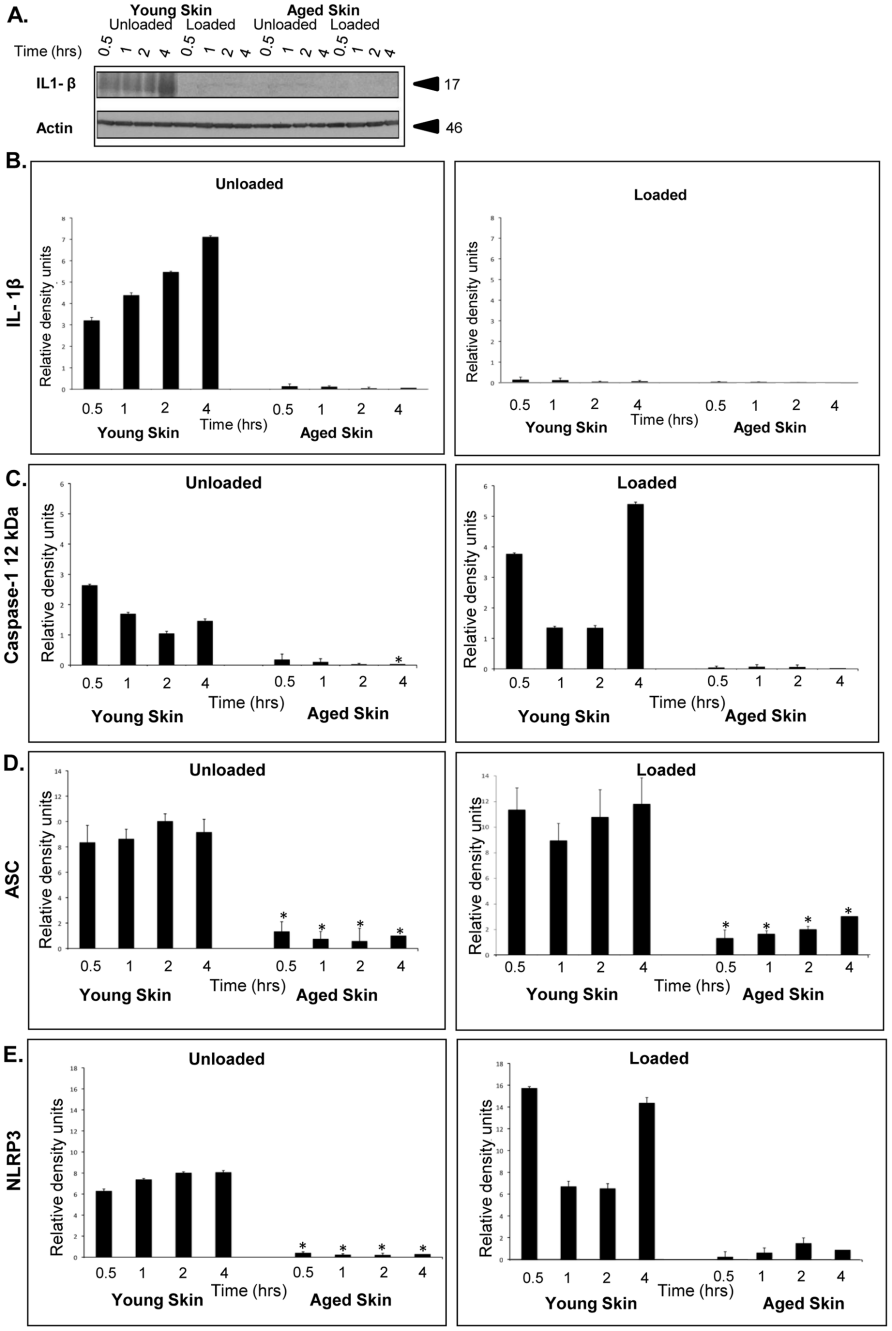
Aged skin has significantly lower levels of IL-1β as compared to young skin regardless of loading, whereas loading induces NLRP3 and caspase-1 protein levels in young human skin. IL-1β levels in loaded and unloaded young and aged skin was determined by Western blots (**A**). Quantification of these immunoblots by densitometry using β-actin as a control (n = 3) (**B**) shows that loading decreased IL-1β expression in young skin. Levels of IL-1β in aged skin are negligible as compared to young skin regardless of load. Levels of NLRP3 inflammasome proteins quantified by Western blot are significantly lower in aged skin as compared to young skin. Caspase-1(**C**), ASC (**D**) and NLRP3(**E**) protein levels, quantified by densitometry using β-actin as a control (n = 3), show that loading induced a significant increase in NLRP3 and caspase-1 expression in young skin 0.5 and 4 hrs upon loading, but not in aged skin. Values are expressed as mean ± SEM. * Indicates p<0.05 by Student t-test.

IL-1β is present and released from epidermal keratinocytes upon UV-radiation, sterile skin trauma and injury [20], [37], [38]. Therefore, we performed western blot analysis to determine IL-1β protein levels. As shown in **Figure 3 (A–B)**, young human skin *ex vivo* expressed the active form of IL-1β, whereas aged skin samples showed barely detectable levels. In response to load, IL-1β decreased only in young whereas it remained low in aged skin, suggesting a decreased inflammatory response to loading and ischemia.

### NLRP3 inflammasome proteins levels are decreased in aged skin

To determine inflammasome protein expression levels in human skin of the two age groups we quantified components of NLRP3 inflammasome complex by immunoblotting. As shown in **Figure 3 (C–E)**, NLRP3 inflammasome components were differentially expressed in young and aged skin. Specifically, caspase-1(**Figure 3C**), ASC (**Figure 3D**) and NLRP3 (**Figure 3E**) were significantly lower in aged skin when compared to young skin. The higher levels of NLRP3 inflammasome proteins in young skin may indicate that aging alters the innate immune inflammatory response.

### Load does not alter NLRP3 inflammasome proteins levels in aged skin

To test whether load alters levels of NLRP3 inflammasome proteins, we quantified NLRP3, ASC and caspase-1 in specimens of both age groups by immunoblotting. As shown in **Figure 3 (C–E)**, load did not alter NLRP3 inflammasome proteins in aged skin. In contrast, in young skin, loading significantly increased levels of NLRP3 inflammasome proteins in a time-dependent manner. After exposure to 0.5 hr of load, caspase-1 and NLRP3 levels significantly increased. These protein increases were maintained for up to 4 hrs of load. No changes in the levels of ASC were observed. However, 4 hr continuous load induced levels of caspase -1 and NLRP3 in young skin.

## Discussion

**Figure 4:**
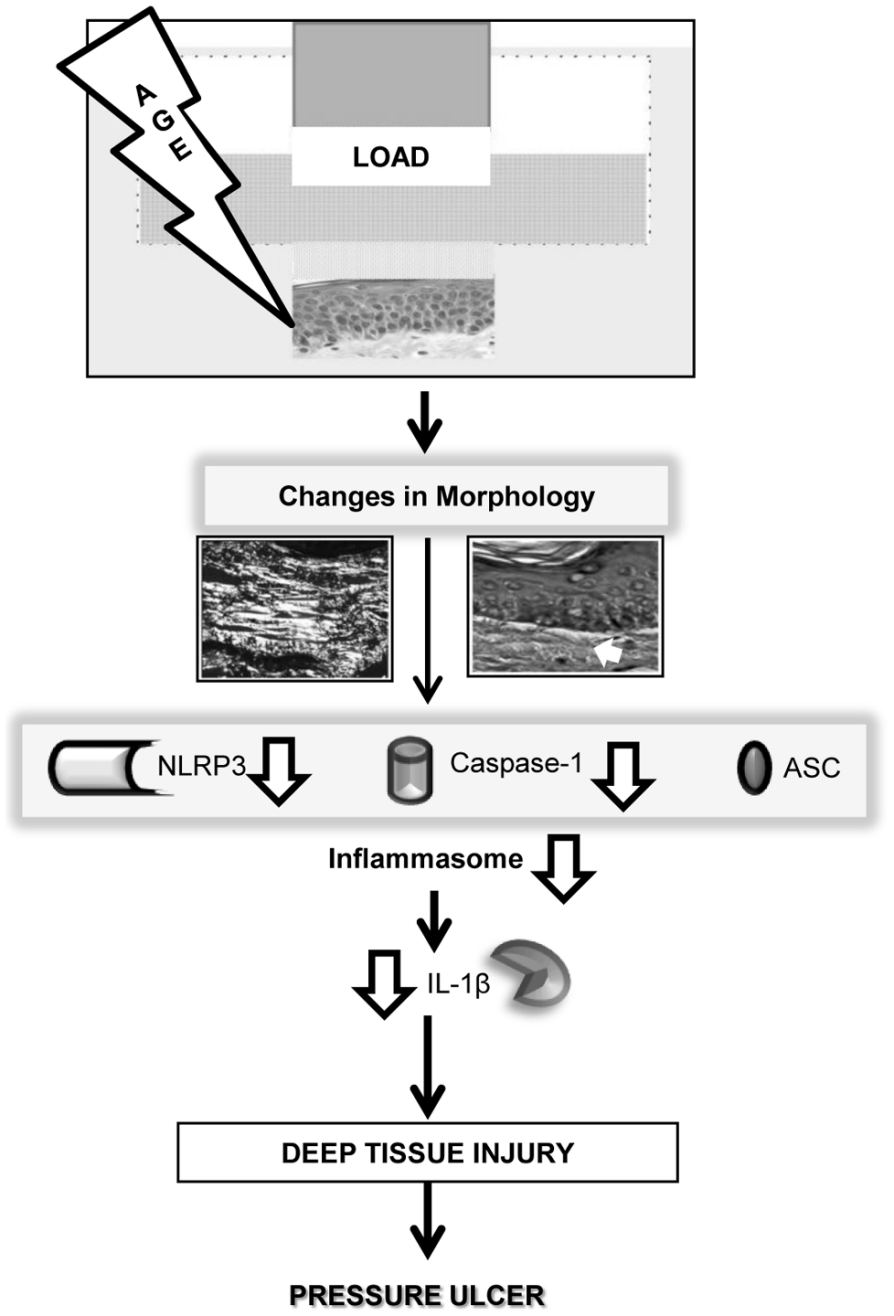
Diagram summarizes effects of load and aging in development of deep tissue injury and pressure ulcers.

In this report, we describe a novel mechanism by which biomechanical load (compressive confined pressure) leads to changes in skin morphology and inflammasome activation as early changes that lead to development of DTI, and possibly PUs. Furthermore, we also describe a novel experimental approach of DTI in human ischemic skin and skin response to confined compressive load/pressure (**Figure 4**). Exposure of human skin to confined compressive load results in significant morphological changes, including subepidermal separation, and altered orientation of collagen fibers. These changes are affected by age and length of exposure. Aged skin showed changes as early as 2 hrs of loading, and changes in collagen alignment were only observed in aged skin. We found that aged skin expressed NLRP3 inflammasome proteins at very low levels and that loading did not alter their expression. Interestingly, we found that loading induced significant alterations in NLRP3 inflammasome protein levels in young skin in a time-dependent manner. Interestingly, extended continuous load induced NLRP3 inflammasome proteins, suggesting that prolonged load may lead to excessive inflammatory response in ischemic young skin. Taken together, these data demonstrate that load/pressure triggers specific tissue response in skin, morphological changes and rapid inflammatory response that may not be entirely preventable. Furthermore, we provide evidence that aging markedly influences response to load, underscoring its role in development of PUs among elderly.

Keratinocytes play a major role in the inflammatory response, a well-tuned tissue response that initiates wound healing process in skin and other tissues [20], [39]–[41]. Inflammasome proteins are synthesized by keratinocytes [37], [42], activated in psoriasis model [23] and IL-1β is released from cells in response to the injury and metabolic stress [43], [44]. NLRP3 induces activation of caspase-1 via interactions with the adaptor protein ASC. Based on our present findings showing presence of NLRP3, ASC and caspase-1 in control young skin, we conclude that human skin *ex vivo* constitutively expresses NLRP3 inflammasome proteins. Similarly, human melanoma cells and central nervous system cells show constitutive levels of inflammasome proteins [45]–[48]. The levels of NLRP3 inflammasome proteins decrease with age, offering possible explanation for decreased immune response in elderly [49]–[51] further supporting the notion that NLRP3 is engaged in the inflammasome signaling in skin. Our studies provide novel findings on how aging and pressure/load regulate inflammasome in skin.

We show that loading of young skin leads to increased levels of NLRP3 and active form of inflammatory caspase-1 (12 kDa), whereas levels of ASC do not change. It appears that initial increase resolves quickly whereas prolonged load (4 hrs) leads to its extended increase. This correlates with morphological changes observed in young ischemic skin at 4 hrs, perhaps suggesting that prolonged load extends inflammasome activation leading to tissue damage. ASC did not show significant change, which is not surprising since it serves as the adaptor protein that brings the NLRP3 and caspase-1 together, which in turn, results in inflammasome activation.

Surprisingly, much lower levels of inflammasome constituents tested were found in aged skin. Loading of aged skin did not cause additional changes in NLRP3 inflammasome proteins. This age-mediated decrease in levels and failure to activate the NLRP3 inflammasome may represent important insights to the skin's innate inflammatory response to load. The mechanism by which the inflammasome regulates wound healing and how aging affects it are under current investigations. Based on our initial observations presented here, coupled with common knowledge that PUs are mostly developed in aged, immobilized individuals [52], [53], [54], we conclude that the inflammasome complex may contribute to pathogenesis of PU.

Inflammatory cytokine IL-1β undergoes maturation and release after cleavage by active caspase-1 [55]. Using METS system we showed that both aging and loading decrease IL-1β levels at every time points studied, suggesting involvement of active IL-1β in pathogenesis of DTI. Decreased levels of IL-1β in aged skin may be due to an altered inflammatory response and dysfunctional inflammasome activation. These findings are consistent with our data showing that the NLRP3 inflammasome protein levels are low in aged skin, regardless if the skin was loaded or not. One can argue that aging may lead to defective IL-1β processing, whereas loading may lead to abolition or delayed IL-1β activation. In young loaded skin the inflammasome protein levels were high but IL-1β levels were low after load, which may suggest that load disrupts the interactions between the inflammasome proteins and inhibits processing of IL-1β even if inflammasome proteins are present. It has been shown, in other systems for instance, that IL-1β can undergo caspase-1 independent activation [56]–[58] and that caspase-1 inhibitors are not able to interfere with the whole spectrum of IL-1 β production [59]. This could explain why the levels of inflammasome proteins remained high in young loaded skin but levels of mature IL-1β (17 kDa) was low. On the other hand, one should keep in mind that reports from studies showed elevated levels of IL1, TNF, MMP's and defensins in human PU [8]–[15], which appears to be in contrast to the findings described here of decreased IL-1 β production in aged skin after loading. The ability to quickly respond to external injury, such as load, should not be interpreted as inability to mount an inflammatory response. In addition, this discrepancy can originate from difference in bacterial presence, which will be included in our future experiments, since presence of infection is one of the frequent problems in PU [60].

In addition to providing new evidence of inflammasome role in development of DTI and underscoring the role of aging in this process, we also describe a model to study initial tissue response to load, early changes that may lead to DTI and further to PU skin response to load. There are number of experimental models that study how mechanical forces affect wound healing [61], though their primary focus was to examine how various devices, such as vacuum-assisted closure or shock-waves, may accelerate wound healing [62], [63], [64], [65]. However, models that utilize application of compressive load to skin to study development of DTI are very scarce. Current animal models include use of magnetic devices [15], [66] or spinal cord injury models [67]. Although important knowledge was gained from these studies, the correlation to human condition is limited, mostly because of anatomical difference between rodents and humans and limitations in precise control of the applied load/pressure. Conversely, early tissue changes leading to the development of PU cannot be captured directly from patients because by the time of specimen collection the PU has already advanced. Here we describe a novel experimental model that utilizes METS to apply confined compressive load to human ischemic skin *ex vivo* and describe age-specific response to it. This model does not include vascular supply that represents both limitation and advantage. Although it cannot measure cellular infiltrate and its role in repair, the advantage is that it resembles profound ischemic conditions. Therefore, the development of the model that utilizes human skin, applies confined compressive load that reflects actual pressure on skin in sacral area of a patient in reclining position and controls the loading time represents very useful and relevant new experimental approach to study mechanisms of DTI/PU development and complements both animal models and patients' biopsies approaches.

It has been shown *in vitro* that small loads cause structural changes to the dermis [68], [69]. Histological changes observed in patients suffering from different stages of PUs [68], [69] showed that the earliest signs of damage appear in the upper dermis. Subepidermal separation and subepidermal bullae occurring without epidermal damage were also found [68], which is strikingly similar to the morphological changes we detected, supporting the value of the model. We report that a continuous load of 300 kPa leads to subepidermal separation in *ex vivo* human skin upon loading for 4 hrs in both, aged and young ischemic skin, suggesting that tissue damage may not be entirely preventable. Changes occur more rapidly in aged skin and alterations in collagen fibers orientation are feature of aged skin. Thinning of dermis, reduced collagen production, increased MMP-1 and slow proliferation of fibroblast in aged skin [70]–[74] are contributing factors. Our findings suggest that compressive load further affects tissue collagen fiber orientation. This added damage to the matrix may contribute to additional decrease in stiffness of dermis, making skin more susceptible to PU development with age, supporting clinical observations of high incidence of PU in elderly [52], [53].

Taken together, although external load/pressure is viewed as one of the key factors in the development of DTI/PU, observed decrease in NLRP3 inflammasome proteins in aged skin may indicate diminished innate immune response in skin due to aging, providing an explanation why PU develop more frequently in elderly. These findings coupled with the changes in skin morphology, collagen orientation and molecular composition of skin underscore the role of aging and compressive load in the pathogenesis of DTI. Our findings may provide important new insights for development of treatment strategies for patients with DTI that may be helpful for future preventions of PU formation among individuals at risk.
